# Risk factors of keloids in Syrians

**DOI:** 10.1186/s12895-016-0050-5

**Published:** 2016-09-20

**Authors:** Abeer Shaheen, Jamal Khaddam, Fadi Kesh

**Affiliations:** 1 Department of dermatology, Tishreen University, Lattakia, Syria; 2 Department of Plastic and Reconstructive Surgery, Tishreen University, Lattakia, Syria

**Keywords:** Keloids, Risk factors, Blood groups, Cause of scarring, Anatomical site, Single site, Multiple sites, Family history, Age of onset, Syrians

## Abstract

**Background:**

Keloid is a benign fibrous growth, which presents in scar tissue of predisposed individuals. It is a result of irregular wound healing, but the exact mechanism is unknown. However, several factors may play a role in keloid formation. To date, there are no studies of keloids in Syria, and limited studies on Caucasians, so we have investigated the risk factors of keloids in Syrians (Caucasians), and this is the main objective of this study.

**Methods:**

Diagnosis of keloids was clinically made after an interview and physical examination. We did a histopathological study in case the physical examination was unclear**.**

The following information was taken for each patient; sex, Blood groups (ABO\Rh), cause of scarring**,** anatomical sites, age of onset, number of injured sites (single\multiple) and family history.

**Results:**

We have studied the clinical characteristics of 259 patients with keloids,130 (50.2 %) females and 129 (49.8 %) males**.** There were 209 (80.7 %) patients with keloids in a single anatomical site compared to 50 (19.3 %) patients with 130 keloids in multiple anatomical sites, 253 (97.68 %) patients with keloids caused by a single cause for each patient compared to 6 (2.32 %) patients with keloids caused by two different causes for each patient.

Keloids could follow any form of skin injury, but burn was the most common (28.68 %). Also, keloids could develop at any anatomical sites, but upper limb (20 %) followed by sternum (19.17 %) was the most common. Over half of the patients developed keloids in the 11–30 age range. 19.3 % (50/259) of patients had family history, 76 % (38/50) of them had keloids located in the same anatomical sites of relative, also, 66 % (33\50) of them had keloids caused by the same cause.

The following information was found to be statistically significant; people with blood group A (*p =* 0.01) compared with other blood groups, spontaneous keloids in patients with blood group A (*p =* 0.01), acne in males (*p =* 0.0008) compared to females, acne in someone who has a previous acne keloid (*p =* 0.0002), burn in someone who has a previous burn keloid (*p =* 0.029), family history, especially for spontaneous (*p =* 0.005), presternal (*p =* 0.039) and shoulder (*p =* 0.008) keloids, people in second and third decades (*p =* 0.02) (*p =* 0.01) respectively.

**Conclusion:**

Age of onset, sex, cause of scarring, blood groups, anatomical site, presence of family history and the number of site (multiple\single) were significant in keloid formation in Syrians.

## Background

Keloid is a benign fibrous growth, presents in scar tissue of predisposed individuals, extends beyond the borders of the original wound, doesn’t usually regress spontaneously, and tends to recur after excision [1]. It is a result of irregular wound healing [2, 3], but the exact mechanism is unknown [1].

There is a clear genetic component given the correlation with family history, high occurrence in identical twins [4, 5], higher predisposition in Blacks, Hispanics and Asians, less frequently in Caucasians and rarity in albinos [1, 4, 6, 7]. Proposed inheritance patterns include autosomal recessive [1], autosomal dominant [1–3, 8, 9] with incomplete penetrance, and variable expression. Maybe the heredity of certain antigens like (HLA-B14, HLA-B21, HLA-Bw16, HLA-Bw35, HLA-DR5, HLA-DQw3, and blood group A) [1] is the underlying reason of genetic predisposition. Genetically susceptible individuals form keloids after wounding but not at every body site, and not after all insults of skin [4], which suggest the effect of both anatomical site and form of skin injury in keloid formation. Although keloids can occur at any age, they are most likely to occur between the ages of 11 and 30 years, which demonstrates the importance of age in keloid formation [8]. Also, keloid growth may be stimulated by sexual hormones due to the higher incidence of keloid formation during puberty and pregnancy, and remarkable decrease after menopause [10].

To date, there are no studies of keloids in Syria, and limited studies on Caucasians, so we investigated the risk factors of keloids in Syrians (Caucasians), and this is the main objective of this study.

## Methods

This study was conducted between March/2013 and August/2015 in the departments of dermatology, at Tishreen and Alassad Hospitals, Lattakia, Syria.

The diagnosis of keloids was clinically made after an interview and physical examination. We did a histopathologic study if the physical examination was unclear, especially for genitalia, buttock, palm and sole keloids, and for other uncertain scars.

### Inclusion/exclusion criteria

All patients with keloids were included into this study, while patients with hypertrophic scare were excluded. Hypertrophic scars are defined as raised scars that remained within the boundaries of the original lesion, often regressing spontaneously after the initial injury and rarely recurring after surgical excision. In contrast, a keloid scar is defined as a dermal lesion that spreads beyond the margin of the original wound, continues to grow over time, does not regress spontaneously and commonly recurring after excision [4].

### Data collection

The following information was taken for each patient: Sex (male/female), blood groups (ABO and Rh), cause of scarring or form of skin injury (which was divided into 7 causes:, burn, surgical wound, sharp wound or knife laceration and not surgical, trauma or laceration, acne, unknown (spontaneous) and other), the age of onset (which was divided into seven age groups: 0–10, 11–20, 21–30, 31–40, 41–50, 51–60 and >60), anatomical sites (face, neck, scalp, earlobe, pinna, upper limb, lower limb, shoulder, lower back, sternum, chest wall without sternum, abdominal wall, palm and sole, genitalia and buttock), the number of injured anatomical sites (single/multiple), family history, the anatomical site and the cause of relative when family history is positive.

**Note:** Multiple sites refer to keloids found in a multiple number of anatomical sites as opposed to multiple keloids found in the same anatomical site. Therefore, the presence of more than one scar in the same anatomical site was not considered to be multiple keloids. Also, a single site keloid refers to a keloid or a number of keloids found in only one anatomical site.

### Control group

We have to compare some of our data (blood groups, sex and age groups) with similar society data, so we got the relative frequency of blood groups from National Blood Transfusion Center (NBTC) of Lattakia for 2015, and the relative frequency of sex and age groups from the Syrian Census Center (SCC) for 2011, which was the last data of SCC because of war conditions. We needed chi-square test to compare that data, so we created a miniature group containing 259 people reflecting society data (blood groups, sex and age groups). We considered it as a control group.

### Statistical analysis

Microsoft Excel 2012 and Microsoft Word 2012 were used for tables and figures. Calculation for the chi-square test on http://www.quantpsy.org/chisq/chisq.htm was used for comparing data. A *P*-value of less than 0.05 was considered statistically significant.

## Results

We studied the clinical characteristics of 259 patients with keloids,130 (50.2 %) females and 129 (49.8 %) males. There were 209 patients with keloids in single anatomical site compared to 50 patients with 130 keloids in multiple anatomical sites, and 253 patients with keloids caused by a single cause for each patient compared to 6 patients with keloids caused by two different causes for each patient. According to that, we had 259 patients with 339 keloids spread over 15 anatomical sites, and 265 causes of scarring as a final result to 7 different forms of skin injury (Table 1). The associations among the age of onset, sex, causes of scarring, blood groups, anatomical sites, presence of family history and number of injured anatomical sites (multiple/single) were analyzed in detail and were statistically evaluated.

**Table 1:** Demographic details of patients with keloids

		Males	Females	Totals	Final total
Frequency of patients (%)		129 (49.8 %)	130 (50.2 %)	259 (100 %)	
Causes	Spontaneous	13	21	34 (12.83 %)	265 (100 %)
	Burn	34	42	76 (28.68 %)	
	Sharp wound	21	20	41 (15.47 %)	
	Surgical	33	32	65 (24.53 %)	
	Trauma	10	10	20 (7.55 %)	
	Acne	21*	5	26 (9.81 %)	
	Others (sting, varicella)	0	3	3 (1.13 %)	
Anatomical sites	Face	13	6	19 (5.6 %)	339 (100 %)
	Neck	14	9	23 (6.78 %)	
	Scalp	8	0	8 (2.36 %)	
	Ear lobe	0	9	9 (2.65 %)	
	Pinna	3	0	3 (0.885 %)	
	Upper limb	34	34	68 (20.06 %)	
	Lower limb	12	14	26 (7.67 %)	
	Shoulder	19	25	44 (12.98 %)	
	Lower back	9	6	15 (4.424 %)	
	Sternum	38	27	65 (19.17 %)	
	Chest wall	11	16	27 (7.96 %)	
	Abdominal wall	9	12	21 (6.19 %)	
	Palm and sole	1	2	3 (o.885 %)	
	Genital	2	2	4 (1.18 %)	
	Buttock	1	3	4 (1.18 %)	
Blood groups	A	62	53	115 (44.4 %)*	259 (100 %)
	B	17	18	35 (13.51 %)	
	AB	6	10	16 (6.2 %)	
	O	44	49	93 (35.9 %)	
	RH +	119	119	238 (91.9 %)	259 (100 %)
	RH−	10	11	21 (8.1 %)	
Age groups	A	18	29	47 (18.15 %)	259 (100 %)
	B	41	39	80 (30.89 %)	
	C	38	32	70 (27.03 %)	
	D	6	16	22 (8.5 %)	
	E	11	9	20 (7.72 %)	
	F	7	4	11 (4.25 %)	
	G	8	1	9 (3.47 %)	
Family history		29	21	50	
F.H in the same site		15	23	38	
F.H by the same cause		16	17	33	
Multiple sites		29	21	50 (19.3 %)	259 (100 %)
Single site		100	109	209 (80.7 %) S	

(*p < 0.05)

Incidence of keloids was equal in females 130 (50.2 %) and males 129 (49.8 %), with statistical significance for developing keloids neither male nor female compared to control (*p =* 0.79) (Table 2). However, males who were older than forty had statistical significance for developing keloids compared to females in the same age (*p =* 0.036) (Table 1).

**Table 2:** Comparison between blood groups, sex and age groups in both groups (patients versus controls)

Society data			Relative frequency of society data (%)	Control group	Frequency of patients	Relative frequency of patients (%)
Blood groups (ABO)	A		34	88	115*	44.4
	B		15.4	40	35	13.51
	AB		8.5	22	16	6.2
	O		42.1	109	93	35.9
Total			100	259	259	100
Blood groups	RH+		91.1	236	238	91.9
RH	RH−		8.9	23	21	8.1
Total			100	259	259	100
sex	Male		51	132	129	49.8
	Female		49	127	130	50.2
Total			100	259	159	100
Age groups Y	A	0–10	25.6	66	47*	18.15
	B	11–20	22.4	58	80*	30.89
	C	21–30	17.6	46	*70	27.03
	D	31–40	12.3	32	22	8.5
	E	41–50	9.3	24	20	7.72
	F	51–60	6.5	17	11	4.25
	G	60<	6.3	16	9	3.47
Total			100	259	259	100

(*P < 0.05)

**Fig. 1:**
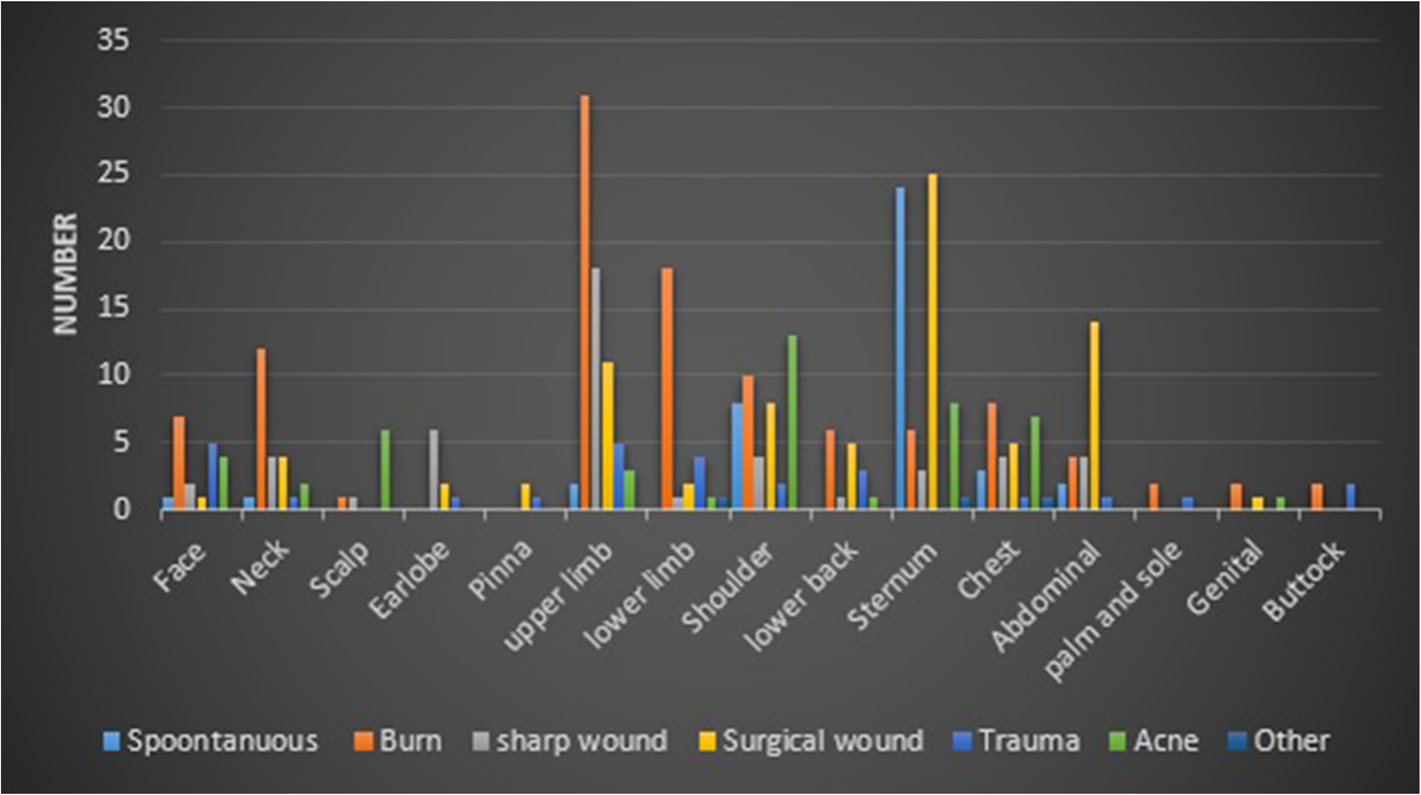
Distribution of causes of scaring according to anatomical sites

Keloids could follow any form of skin injury, but burn was the most common (76\265) (28.68 %), and trauma (20\265) (7.55 %) was the least (Table 1). Causes had almost coordinated distribution in males and females, but males had higher predisposition to develop acne keloids compared to females (*p =* 0.0008) (Table 1). Also, keloids could develop at any anatomical sites, but upper limb (68\339) (20 %) followed by sternum (65\339) (19.17 %) were the most common, while buttock, genitalia, palm and sole were the least. (Table 1). Distribution of causes of keloids according to anatomical sites are demonstrated at (Fig. 1) (Table 3).

**Table 3:** The most common causes of scarring according to anatomical sites, and the most common anatomical sites of keloids according to causes

Causes	The most common anatomical sites (%)	Anatomical sites	The most common causes (%)
Spontaneous	Sternum (58.54 %), shoulder (19.5 %)	Face	Burn (35 %)
Burn	Upper limb (28.44 %), lower limb (16.51 %)	Neck	Burn (50 %)
Sharp wound	Upper limb (37.5 %)	Scalp	Acne (75 %)
Surgical	Sternum (31.25 %), abdominal (17.5 %)	Ear lobe	Sharp wound (66.66 %)
Acne	Shoulder (28.26 %), sternum (17.4 %)	Pinna	Surgical (66.66 %)
Trauma	Face (18.52 %) upper limb (18.52 %)	Upper limb	Burn (44.29 %)
		Lower limb	Burn (66.66 %)
		Shoulder	Acne (28.88 %)
		Lower back	Burn (37.5 %)
		Sternum	Spontaneous (35.82 %) Surgical (37.13 %)
		Chest wall	Burn (27.59 %)
		Abdominal wall	Surgical (56 %)
		Palm and sole	Burn (66.66 %)
		Genital	Burn (50 %)
		Buttock	Trauma (50 %) Burn (50 %)

**Fig. 2:**
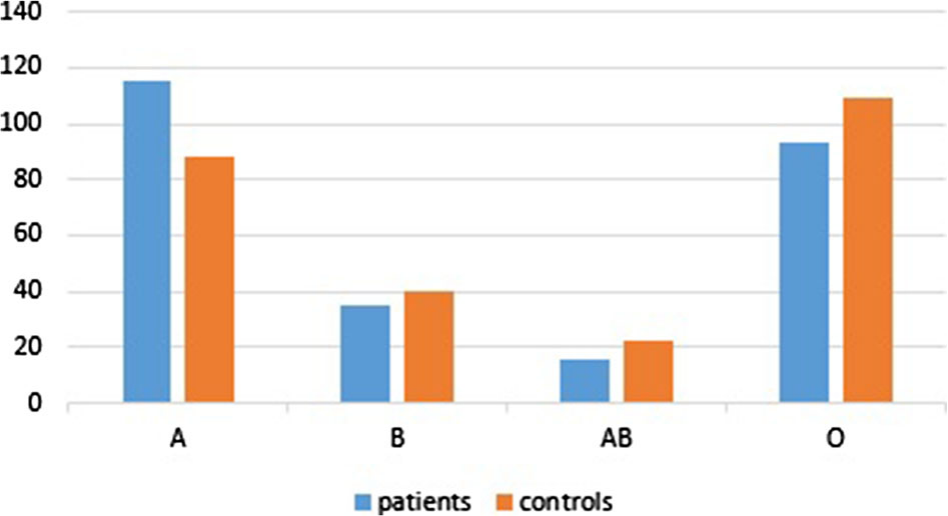
Distribution of Blood groups ABO in patients and controls

Univariate analysis between each blood group in both groups (patients versus controls) found statistical significance for developing keloids in people with blood group A (*p =* 0.01) (Table 2) (Fig. 2), especially for spontaneous keloids (*P =* 0.01) (Table 4). However, the proportion of Rh + subjects wasn’t significantly different between control (91.1 %) and patients (91.9 %) (*p =* 0*.*7) (Table 2).

**Table 4:** The possible effect of blood group A on development of spontaneous keloids

Blood group (N)	Spontaneous keloids		Total
	Yes	No	
Blood group A	22	93	115
Blood groups B, AB, O	12	132	144
Total	34	255	259
*P* value (chi-square test)	0.01		

**Fig. 3:**
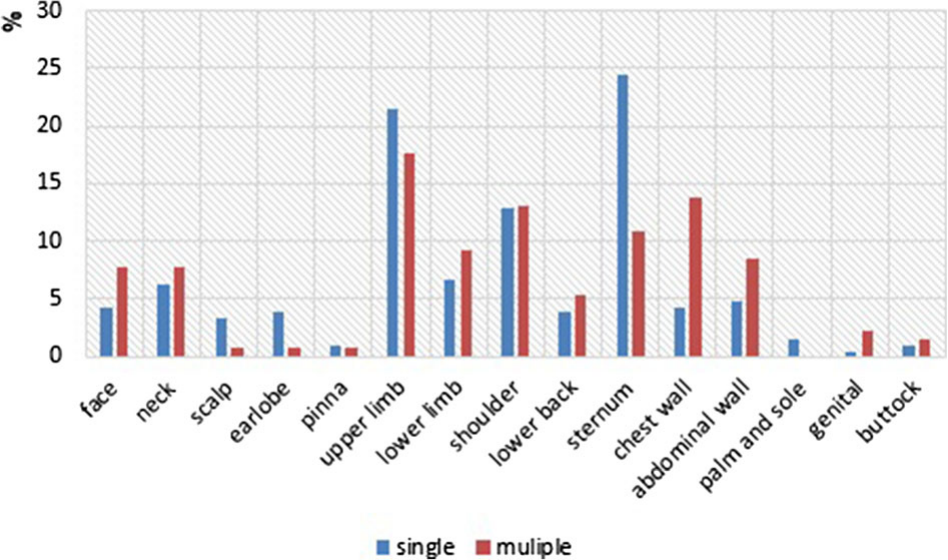
Comparison of single versus multiple site-specific keloids in different anatomical sites

Keloids occurred in patients, either in single or in multiple anatomical sites. 19.3 % (50/259) of patients had 130 keloids in multiple anatomical sites as opposed to 80.7 % (209/259), who had a keloid in a single site (Table 1). The upper limb was the most common site for patients with multiple site keloids (23\130) (17.7 %), and sternum was the most common one for patients with single site (51\209) (24.4 %) (Fig. 3). All causes tended to develop keloids in multiple sites, but burn was the most common (21\55) (38.2 %), while surgical wound was the most common one for patients with a single site (56\210) (26.67 %) (Table 5). There was statistical significance for developing both burn and acne keloids in multiple anatomical sites (*p =* 0.029) (*p =* 0.0002) respectively (Table 6). Also, keloids followed either single or multiple forms of skin injury. Only 2.32 % (6\259) of patients had keloids caused by two different causes for each patient, 5\6 of them had a surgical cause, and 5\6 of them had keloids in multiple sites.

**Table 5:** Distribution of causes of scarring according to (single\multiple) sites

	Spontaneous	Burn	Acne	Trauma	Sharp wound	Surgical	other	Total	Number of patients
Single	29 (13.81 %)	55 (26.19 %)	14 (6.67 %)	15 (7.14 %)	38 (18.1 %)	56 (26.7 %)	3 (1.43 %)	210 (100 %)	209
Multiple	(5) 9.1%	21 (38.2 %)	12 (21.82 %)	5 (9.1 %)	3 (5.45 %)	9 (16.36 %)	0	55 (100 %)	50
Total	34	76	26	20	41	65	3	265	259

**Table 6:** Distribution of burn and acne keloids according to (single\multiple) sites

	Burn		Acne		Total
	Yes	No	Yes	No	
Single site	55	154	14	195	209
Multiple sites	21	29	12	38	50
Total	76	183	26	233	259
*P* value (chi-square test)	0.029		0.0002		

**Note:** When we want to compare variables with causes or anatomical sites, we compare with number of patients (259) and not with number of causes (265) or number of sites (339)). As at (Table 5) (Table 6).

**Fig. 4:**
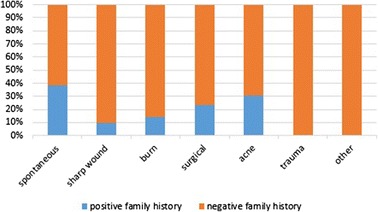
Relative frequency of the causes of scarring according to family history

**Fig. 5:**
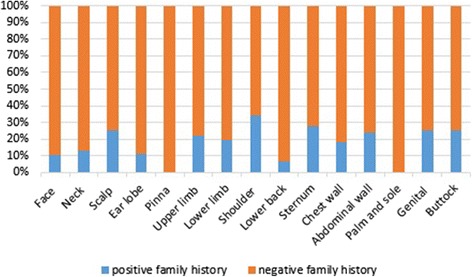
Relative frequency of anatomical sites of keloids according to family history

19.3 % (50/259) of patients had family history, 76 % (38/50) of them had keloids located in the same anatomical sites of relative. Also, 66 % (33\50) of them had keloids caused by the same cause (Table 1). Most of the causes and anatomical sites tended to be inherited, but in different proportions (Fig. 4) (Fig. 5). Patients with spontaneous and acne keloids had the highest percentage of family history (38.23 %) (30.77 %) respectively in contrast to patients with sharp wound keloids, who had the least percentage (9.76 %) (Fig. 4). Also, patients with shoulder and sternum keloids had the highest percentage of family history (34.1 %) (27.7 %) respectively (Fig. 5). We found statistical significance for heredity spontaneous, presternal and shoulder keloids (*p =* 0.002) (*p =* 0.047) (*p =* 0.006) respectively (Table 7).

**Table 7:** Remarkable hereditary of spontaneous, sternum and shoulder keloids

Family history	Spontaneous		Sternum		Shoulder		Total
	Yes	No	Yes	No	Yes	No	
Positive	13	37	18	32	15	35	50
Negative	21	188	47	162	29	180	209
Total	34	225	65	194	44	215	259
*P* value (chi-square test)	0.002		0.047		0.006		

**Fig. 6:**
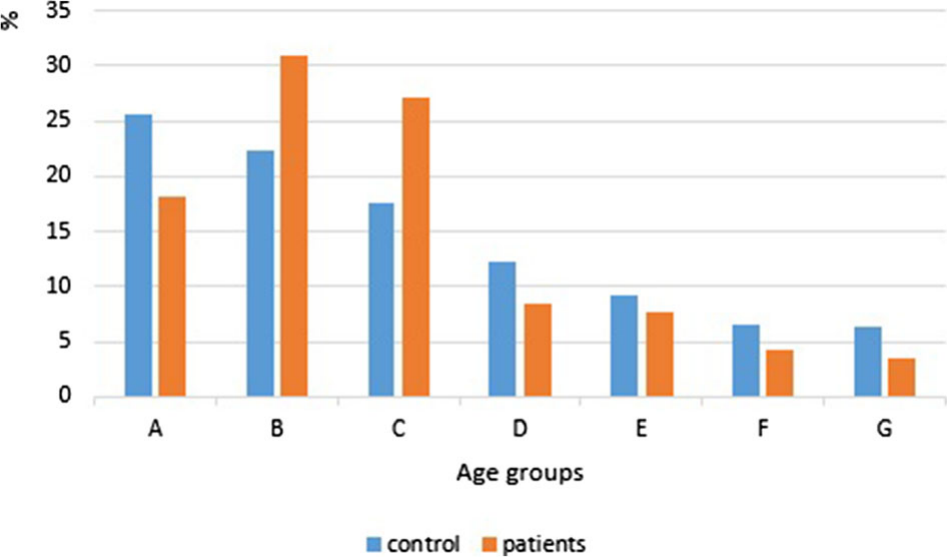
Relative frequency of age groups in patients and control

Over half of the patients developed keloids in the 11–30 age range (Table 1) (Fig. 6).

Both age groups B and C (second and third decades) had statistical significance for developing keloids compared with control (*P =* 0.02) (*P =* 0.01) respectively, as opposed to age group A (first decade), that had statistical significance for not developing keloids compared with control (*p =* 0.04). (Table 2) (Fig. 6).

The distribution of causes and anatomical sites according to age groups were demonstrated at (Table 8) (Table 9) respectively. We have analyzed the relationship between each cause and each anatomical site according to 7 age groups. Then we selected the results that had statistical significance (Tables 10, 11, 12, 13 and 14).

**Table 8:** Distribution of causes of scarring according to age groups

Age groups	Causes								
	spontaneous	Burn	acne	trauma	Sharp wound	Surgical wound	others	Total	Number of patients
A	1	38	0	4	2	2	0	47	47
B	13	13	14	5	21	15	2	83	80
C	11	15	11	6	14	15	1	73	70
D	6	5	0	3	2	6	0	22	22
E	3	2	1	1	0	13	0	20	20
F	0	1	0	1	1	8	0	11	11
G	0	2	0	0	1	6	0	9	9
Total	34	76	26	20	41	65	3	265	259

**Table 9:** Distribution of anatomical sites of keloids according to age groups

Anatomical sites	Age groups							
	A	B	C	D	E	F	G	Total
Face	3	10	5	0	1	0	0	19
Neck	5	7	6	3	2	0	0	23
Scalp	0	2	6	0	0	0	0	8
Ear lobe	0	7	1	0	0	1	0	9
Pinna	1	2	0	0	0	0	0	3
Upper limb	17	14	24	5	5	1	2	68
Lower limb	12	6	2	2	4	0	0	26
Shoulder	5	19	13	4	1	2	0	44
Lower back	4	6	3	1	1	0	0	15
Sternum	4	17	15	7	8	7	7	65
Chest wall	5	8	8	3	2	1	0	27
Abdominal wall	2	7	5	3	3	1	0	21
Palm and sole	2	0	1	0	0	0	0	3
Genital	1	2	0	0	1	0	0	4
Buttock	2	0	1	1	0	0	0	4
Total	63	107	90	29	28	13	9	339
Number of patients	47	80	70	22	20	11	9	259

**Table 10:** Spontaneous and burn keloids in age group A compared with other age groups

Age group	Spontaneous		Burn		Total
	No	Yes	No	Yes	
A	46	1	9	38	47
B,C,D,E,F,G	179	33	174	38	212
Total	225	34	183	76	259
*P* value (chi-square test)	0.01		<0.0001

**Table 11:** Acne, earlobe and sharp wound keloids in age group B compared with other age groups

Age group	Earlobe		sharp wound		Acne		Total
	No	Yes	No	Yes	No	Yes	
B	73	7	59	21	66	14	80
A,C,D,E,F,G	177	2	159	20	167	12	179
Total	250	9	218	41	233	26	259
*P* value (chi-square test)	0.002		0.002		0.008

**Table 12:** Scalp keloids in age group C compared with other age groups

Age group	Scalp keloids		Total
	No	Yes	
C	64	6	70
A,B,E,F,G	187	2	189
Total	251	8	259
*P* value (chi-square test)	0.002

**Table 13:** Distribution of surgical keloids and other keloids (keloids caused by other causes) according to age groups G, F and G

Cause of keloid	Age group E		Age group F		Age group G		Total
	No	Yes	No	Yes	No	Yes	
Surgical keloid	52	13	57	8	59	6	65
Other keloids	187	7	191	3	191	3	194
Total	239	20	248	11	250	9	259
*P* value (chi-square test)	0.003		0.0002		0.00002

**Table 14:** Distribution of presternal keloids and other keloids (keloids in other anatomical sites) according to age groups G and F

Anatomical site of keloid	Age group F		Age group G		Total
	No	Yes	No	Yes	
Presternal keloid	58	7	58	7	65
Other keloids	190	4	192	2	194
Total	248	11	250	9	259
*P* value (chi-square test)	0.003		0.0002		

This mean for each variable (each cause or each anatomical site) we have 7 independent tests. Therefore, because of multiple tests, we lowered the *p* value (to be less or equal 0.01) to reduce false positive.

In age group A (0-10Y), there was statistical significance for developing burn keloids (*p <* 0.0001), and for no developing spontaneous keloids (*p =* 0.01) (Table 10). Upper limb (26.98 %) followed by lower limb (19.05 %) were the most common anatomical sites for developing keloids in this age group.

In age group B (11–20Y), causes had almost coordinated distribution. The shoulder (17.76 %) followed by sternum (15.88 %) were the most common sites for developing keloids in this age group. There was statistical significance for developing sharp wound, acne and earlobe keloids (*p =* 0.002) (0.008) (*p =* 0.002) respectively (Table 11).

In age group C (21–30Y), there was also a coordinated distribution of causes.

The upper limb (26.7 %) followed by sternum (16.7 %) were the most common sites. We found statistical significance for developing scalp keloids in this group (*p =* 0.002) (Table 12).

In age group D (31–40Y), causes had almost coordinated distribution considering the absence of acne keloids. The sternum (24.14 %) was the most common site for developing keloids in this age group without statistical significance.

In age groups E, F and G (41–70 Y), there was statistical significance for developing surgical keloids in these groups (*p =* 0.00002) (*p =* 0.0002) (*p =* 0.003) respectively (Table 13).

The sternum was the most common site for developing keloids in these groups (28.6 %) (53.8 %) (77.8 %) respectively with statistical significance for developing presternal keloids only in F and G groups (*p =* 0.003) (*p =* 0.0002) respectively (Table 14).

**Fig. 7:**
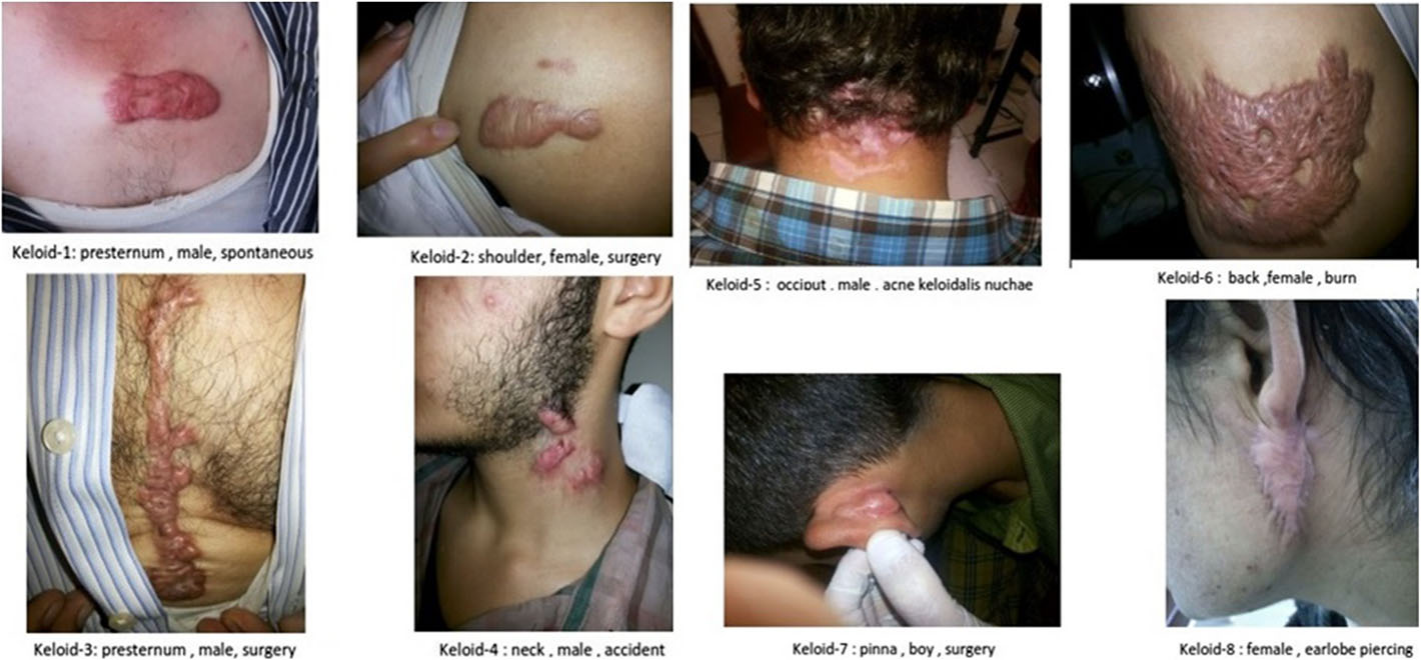
Some keloids of our patients which located in different sites and caused by different forms of skin injury

There were some pictures of keloids of our patients at Fig. 7.

## Discussion

The exact mechanism of keloid formation is unknown, but several factors play a significant role in its formation. The genetic predisposition is the most important factor; other factors are the anatomical site, the form of skin injury, the age of onset and sex. In this study we have analyzed the associations among the age of onset, sex, the cause of scarring, blood groups, the anatomical site, presence of family history and the number of injured anatomical sites (multiple/single) to demonstrate some of the risk factors of keloids in Syrians (Caucasians).

Incidence of keloids was equal in females and males. That agree with most previous studies [5, 6, 11–13]. However, some studies found higher incidence of keloids in females [1, 4], while others found higher incidence in males [14].

Burn was the most common cause of keloid formation in our study (28.68 %), but laceration was the most common one in a Jamaican study [4]. We can explain this finding particularly because of war conditions in our country. The occurrence of acne keloids was higher in males compared to females (*p =* 0.0008), because only males have acne keloidalis nuchae. Also, the severity of acne is higher in them [15].

The most common anatomical site for developing keloids differs according to race, traditions and conditions of study’s society, generally keloids tend to occur on highly mobile sites with high tension [16, 17]. In our study, upper limb (20 %) followed by sternum (19.17 %) were the most common sites for developing keloids, which was similar to a study of dark skin patients [11] (sternum 28.95 %, upper limb 15.8 % and head 16.7 %), and contrary to the Jamaican study [4] (ear 23 %), and Indian one [5] (sternum 33.6 %). On other hand, genitalia (1.18 %), buttock (1.18 %), palm and sole (0.9 %) were the rarest sites in our study, which agree with the previous studies [10, 14].

People with blood group A have high probability to develop keloids compared with other blood groups (*p =* 0.01), that may be partly explained by the association between the effect of red cell antigens A (which present on the membrane surface of red blood cells and certain epithelial cells [11]) and other factors in these patients. Our findings agree with a previous study [5], and disagree with another one [11]. Spontaneous keloid is a rare condition, and it is controversial whether it is in fact spontaneous. The scar tissue may form after an insignificant inflammatory reaction or injury which the patient has no recollection of [18]. However, we found 13.4 % spontaneous keloids, which was similar to Togo study [11] (13.13 %), but lesser than an Iraqi one [13] (34 %). Also, we found statistical significance association between spontaneous keloids and blood group A (*p =* 0.01), which has not been previously reported. This result confirms the effect of red cell antigens A in development of keloids, as we discussed before.

Few studies discussed the development of keloids in single versus multiple anatomical sites [4, 19]. There were 19.3 % of patients who had keloids in multiple anatomical sites, while this percentage was 42 % in the Jamaican study [4]. All causes tended to develop keloids in multiple sites, but only burn and acne had statistical significance (*p =* 0.029) (*p =* 0.0002) respectively, because both acne and burn could affect multiple sites more than other causes, which is more located. This means there was high probability to develop acne or burn keloids in another anatomical site in a patient who had a previous acne or burn keloid respectively. In our study, the upper limb was the most common anatomical site for developing keloids in patient with multiple sites (46 %), and sternum was the most common one in patients with a single site (26.84 %), while the earlobe was the most common site in both multiple (24 %) and single (48 %) sites in the Jamaican study [4]. Also, burn was the most common cause in patients with multiple sites (42 %), and surgical wound was the most common one in patients with a single site (26.8 %), while ear piercing was the most common cause in both sites in that study [4]. We didn’t find any relationships between the development of keloids in multiple anatomical sites and the age of onset, the sex of patient or the presence of family history, while other studies [4, 19, 20] found statistically significant associations among these variables.

Only (6/259) (2.32 %) of our patients had keloids caused by two different causes, compared to 15.9 % of patients in another study [11]. This result confirms the importance of the form of skin injury in keloid formation (not all insults lead to keloids even in the susceptible individuals [4]). (5\6) of these patients had surgical keloids, so we have to be careful when performing surgery for a patient who had a previous keloid. Only 5\259 (1.93 %) of patients had keloids caused by different causes, and distributed on multiple anatomical sites. This means there were only few people who have a high predisposition to develop keloids.

Most keloids occur sporadically, but some cases are familial, 19.3 % of our patients had family history, which was lower than previous studies [4, 14]. 76 % (38/50) of them had keloids located in the same anatomical sites of the relative. Also, 66 % (33\50) of them had keloids caused by the same cause. There was statistical significance for heredity spontaneous keloids (*p =* 0.002), which usually appears in the second decade (there was statistical significance for not developing spontaneous keloids in first decade (*p =* 0.01)) (Table 8), and for heredity presternal and shoulder keloids (*p =* 0.047), (*p =* 0.006) respectively. These results reflect the importance of the cause and anatomical site in heredity of keloid. Some recent studies confirmed the hereditary of spontaneous keloids [19, 21]. Other studies pointed to familial patterns of keloid distribution [9]. We didn’t find any relationships between the family history and sex or the number of sites (single/multiple), while there were statistically significant associations between these variables in other studies [4, 19, 20].

Although keloids could occur at any age, they were rare in first decade (age group A) (*p =* 0.04), because people in this decade are not stimulated by sexual hormones (higher incidence of keloid formation during puberty) [10], most likely to occur in second and third decades (age groups B and C) (*P =* 0.02) (*P =* 0.01) respectively, and tend to decrease in older (Fig. 6), because younger people may have a higher frequency of trauma and their skin is more elastic than the skin of elderly people [22]. These findings agree with many studies [1, 4–6, 19, 20]. Burn was the most common cause for developing keloids in age group A compared with other groups (*p <* 0.0001), especially on upper and lower limbs. This is a logical result, because most of burn accidents exist in younger children especially on extremities. Occurrence of acne keloids was higher in age group B compared with other groups (*p =* 0.008), because the peak in prevalence and severity of acne occurs in second decade [23]. High frequency of sharp wound accidences and earlobe piercing in age group B explain the statistical significance for developing sharp wound (*p =* 0.002) and earlobe (*p =* 0.002) keloids in this age group. Also, development of scalp keloids was higher in age group C compared with other groups (*p =* 0.002), because most cases of acne keloidalis nuchae occur in persons aged 14–25 years [24] (acne keloidalis nuchae caused 75 % of scalp keloids in our study). We noted absence of acne keloids in age group D, because frequency of acne extremely decrease in this age [25, 26]. At last, development of surgical keloids was higher in age groups E, F and G compared with other groups (*p =* 0.00002) (*p =* 0.0002) (*p =* 0.003) respectively, especially on sternum. These results reflect an increase of open heart surgeries in older people, especially for males who were older than forty compared to females in the same age (*p =* 0.036).

**Note:** All patients with multiple site keloids had the same age group for each one.

We can explain this result; Patients with burn keloids were war or extensive burn victims, and patients with trauma and cut wound keloids were war or road accident victims. Patients with surgical keloids had extensive surgical wounds, or multiple surgical wounds which have occurred nearly in same time. As we know, acne keloids followed acne, which usually occur in specific age groups. It is surprising that all patients with multiple spontaneous keloid had the same age group for each one, that may reflect the predisposition for developing spontaneous keloid differs from one to another, or because we didn’t follow up the patient, so we can’t know if they will develop keloid later.

At last, we have demonstrated some of the risk factors of keloids:

- People with blood group A compared with other blood group.
- Spontaneous keloids in patients with blood group A.
- Acne in males compared to females.
- Acne in someone who has a previous acne keloid, because acne keloids tend to develop in multiple sites.
- Burn in someone who has a previous burn keloid, because burn keloids tend to develop in multiple sites.
- Family history, especially for developing spontaneous, presternal and shoulder keloids.
- People in second and third decades.

## Conclusions

It is possible that several factors such as the age of onset, sex, the cause of scarring, blood groups, the anatomical site, the presence of family history and the number of injured sites (multiple/single) have an important role in keloid formation and consequentially in predicting a keloid’s behavior in response to treatment and prognosis. In this study we have demonstrated the significance of the previous factors in keloid formation in Syrian population. Therefore, we have to generalize these results, especially for dermatologists and surgeons. These observations could indicate a genetic basis in keloid formation, which justifies the need for genetic studies and more studies about families with keloids to define the type of heredity in our patients. (We noted that only females in two families (in three generations) had keloid formation. That suggests kind of sex-linked heredity, which has not been previously reported).

## Abbreviations

NBTC: National blood transfusion center
SCC: Syrian census center
